# Establishment of Homozygote Mutant Human Embryonic Stem Cells by Parthenogenesis

**DOI:** 10.1371/journal.pone.0138893

**Published:** 2015-10-16

**Authors:** Silvina Epsztejn-Litman, Yaara Cohen-Hadad, Shira Aharoni, Gheona Altarescu, Paul Renbaum, Ephrat Levy-Lahad, Oshrat Schonberger, Talia Eldar-Geva, Sharon Zeligson, Rachel Eiges

**Affiliations:** 1 Stem Cell Research Laboratory, Shaare Zedek Medical Center affiliated with the Hebrew University School of Medicine, Jerusalem, Israel; 2 Zohar PGD Lab, Medical Genetics Institute, Shaare Zedek Medical Center affiliated with the Hebrew University School of Medicine, Jerusalem, Israel; 3 IVF Unit, Shaare Zedek Medical Center affiliated with the Hebrew University School of Medicine, Jerusalem, Israel; Wellcome Trust Centre for Stem Cell Research, UNITED KINGDOM

## Abstract

We report on the derivation of a diploid 46(XX) human embryonic stem cell (HESC) line that is homozygous for the common deletion associated with Spinal muscular atrophy type 1 (SMA) from a pathenogenetic embryo. By characterizing the methylation status of three different imprinted loci (MEST, SNRPN and H19), monitoring the expression of two parentally imprinted genes (SNRPN and H19) and carrying out genome-wide SNP analysis, we provide evidence that this cell line was established from the activation of a mutant oocyte by diploidization of the entire genome. Therefore, our SMA parthenogenetic HESC (pHESC) line provides a proof-of-principle for the establishment of diseased HESC lines without the need for gene manipulation. As mutant oocytes are easily obtained and readily available during preimplantation genetic diagnosis (PGD) cycles, this approach should provide a powerful tool for disease modelling and is especially advantageous since it can be used to induce large or complex mutations in HESCs, including gross DNA alterations and chromosomal rearrangements, which are otherwise hard to achieve.

## Introduction

Parthenogenesis is the process by which an oocyte is stimulated to divide and develop into an embryo without being fertilized. It can be artificially induced by triggering unfertilized eggs to resume meiosis without undergoing cell division, or achieved spontaneously following failed fertilization. The resulting embryos, which contain only maternal chromosomes, are unviable due to poor development of extra embryonic tissues [1–7]. Yet, they can easily develop into blastocysts which, quite remarkably, can be used to derive maternal-only human embryonic stem cells [8–17]. Parthenogenetic HESCs (termed pHESC) are usually diploid and either homozygous or heterozygous, depending on the oocyte stage at which cell division is interrupted. If meiosis is blocked at metaphase I then the HESC line will be entirely heterozygous; if meiosis is blocked at metaphase II (MII), then the cells will present large blocks of homozygosity; and if cell division is blocked after extrusion of the second polar body, then the cell line will be entirely homozygous [18,19]. Hence, parthenogenesis may be used as a tool to create homozygote affected HESC lines by pharmacological treatment of mutant oocytes from MII and later in more advanced stages. Such oocytes are readily available in in vitro fertilization (IVF) clinics performing preimplantation genetic diagnostic (PGD) procedures. PGD refers to genetic testing of embryos before conception, a procedure that relies on (IVF) treatment and is offered to couples at risk who wish to prevent the birth of genetically diseased children. Here we provide a proof-of-principle for the use of parthenogenesis to establish homozygous mutant HESCs from oocytes retrieved through PGD treatment without the need for genetic manipulation.

## Materials and Methods

This study was approved by the Ethics Committee at Shaare Zedek Medical Center (IRB 87/07) for the derivation of HESC lines from genetically affected PGD derived embryos, in compliance with protocols approved by the National Ethics committee.

### HESC line derivation

The SMA HESC line was established from a PGD derived embryo that developed into a fully grown hatched blastocyst by day 7 following fertilization. At this stage the inner cell mass (ICM) was mechanically isolated by manual cutting with an ultra-sharp splitting blade (Bioniche). The intact ICM clump was placed on a feeder cell layer of mitomycin C-inactivated mouse embryonic fibroblasts and cultured in HESC media (knockout DMEM supplemented with 20% KO-serum replacement, 1% nonessential amino acids, 1mM l-glutamine, 0.5% insulin–transferrin–selenium, 50U/mL penicillin, 50mg/mL streptomycin, 0.1mM beta-mercaptoethanol, and 30 ng/mL bFGF). Outgrowths of proliferating cells were manually propagated using the cut-and-paste method. Following 5–7 passages, the newly established HESC line was further propagated by collagenase type IV (Gibco) and then frozen for future use as described in [20].

### Expression of undifferentiated-specific markers

SMA HESCs were examined for the expression of undifferentiated cell specific markers by immunostaining using Oct3/4 mouse monoclonal antibody (Santa Cruz Biotechnology sc-5279, 1:50 dilution) or TRA-1-60 mouse monoclonal antibody (Santa Cruz Biotechnology sc-21705, 1:50 dilution), together with Cy3-conjugated goat anti-mouse polyclonal antibodies (Jackson Immunostaining, 115-035-062, 1:100 dilution). Nuclear staining was performed with Hoechst 33258 (Sigma, Inc. 861405). Staining for alkaline phosphatase was carried out using an Alkaline Phosphatase Kit (Sigma Diagnostics, Inc. 86R-1KT), according to the manufacturer's protocol.

### RT-PCR

Total RNA was isolated from cells by TRI reagent extraction, then 1μg RNA was reverse transcribed by random hexamer priming and Multi Scribe^™^ reverse transcriptase (ABI). Amplification was performed using the primers listed in S1 File with Supertherm^™^ Taq DNA polymerase (Roche).

### Chromosome analysis

Karyotype analysis was carried out by Giemsa staining according to standard protocol.

### *SMN1* Genotyping by PCR-restriction, MLPA, and haplotype analyses

Testing for the presence/absence of *SMN1* was carried out using PCR-restriction, multiplex ligation-dependent probe amplification (MLPA), and haplotype analyses. For PCR-restriction, the DraI endonuclease was used to detect homozygous deletion of exon 7 in *SMN1* according to [21](see S1 File for PCR primer sequences used for the assay). Assessments of copy number variations in *SMN1* were carried out using a commercial MLPA kit (SALSA MLPA P060-B1, MRC Holland) according to the manufacturer’s protocol. For haplotype confirmation, the primer pairs and fluorescent PCR conditions listed in S1 File were used to amplify 5 different *SMN1*-flanking microsatellites which were separated according to size by capillary electrophoresis.

### Teratoma induction

2.5-5X10^6^ cells were harvested, diluted 1:1 in media:Matrigel and injected subcutaneously to both sides of the back of NOD-SCID IL2R-/- mice. 6–8 weeks later, the mice were sacrificed and two independently induced tumors were isolated, sectioned and assessed for differentiation by H&E staining.

### Bisulfite Sequencing

Genomic DNA (2μg) was modified by bisulfite treatment (EZ DNA methylation Kit^TM^, Zymo Research) and amplified by FastStart^™^ DNA polymerase (Roche) using primers listed in S1 File. Amplified products were cloned and single colonies were analyzed for CpG methylation by direct sequencing (ABI 3130XL). For pyrosequencing, PCR products were analyzed using PyroMak Q24 (QIAGEN).

### Affymetrix CytoScan 750K Array

DNA was extracted from a peripheral blood sample and a cell line using the QiaAmp DNA Mini Kit (Qiagen) and analysed on the Affymetrix^®^ CytoScan 750K Array according to the manufacturer's protocol (Affymetrix, Santa Clara, CA). The CytoScan 750K Array provides whole-genome coverage, with more than 750,000 markers for copy number analysis and over 200,000 SNPs for genotyping. Data analysis was performed using the Chromosome Analysis Suite (CHAS) (Affymetrix, Santa Clara, CA). All markers were used to determine copy number and SNP probes were used to calculate loss of heterozygosity (LOH) and genotype calls. Microarray data from this study can be accessed at GEO accession number GSE72284.

## Results

We report on the derivation of a parthenogenetic HESC (pHESC) line that is affected with spinal muscular atrophy type 1 (SMA1, OMIM #253300), an infantile progressive muscular atrophy with autosomal recessive inheritance. Nearly 95% of SMA patients carry a homozygous deletion of the *SMN1* gene, which resides within the *SMN* locus (5q13.2)[22]. The *SMN* locus is composed of a 500kb inverted duplication in which deletions and gene conversions frequently arise in two highly homologous genes named *SMN1* and *SMN2*. These closely related genes are distinguished by just two nucleotide alterations; one in exon 7 and one in exon 8. The single nucleotide difference in exon 7 of *SMN2* is responsible for alternative splicing, with skipping of exon 7 in the mature transcript of *SMN2*. This leads to the formation of a truncated SMN protein that is less stable and incapable of compensating for the activity of the full-length protein that is normally generated by *SMN1*. This full length protein is essential for the survival and maintenance of motor neurons.

**Fig 1:**
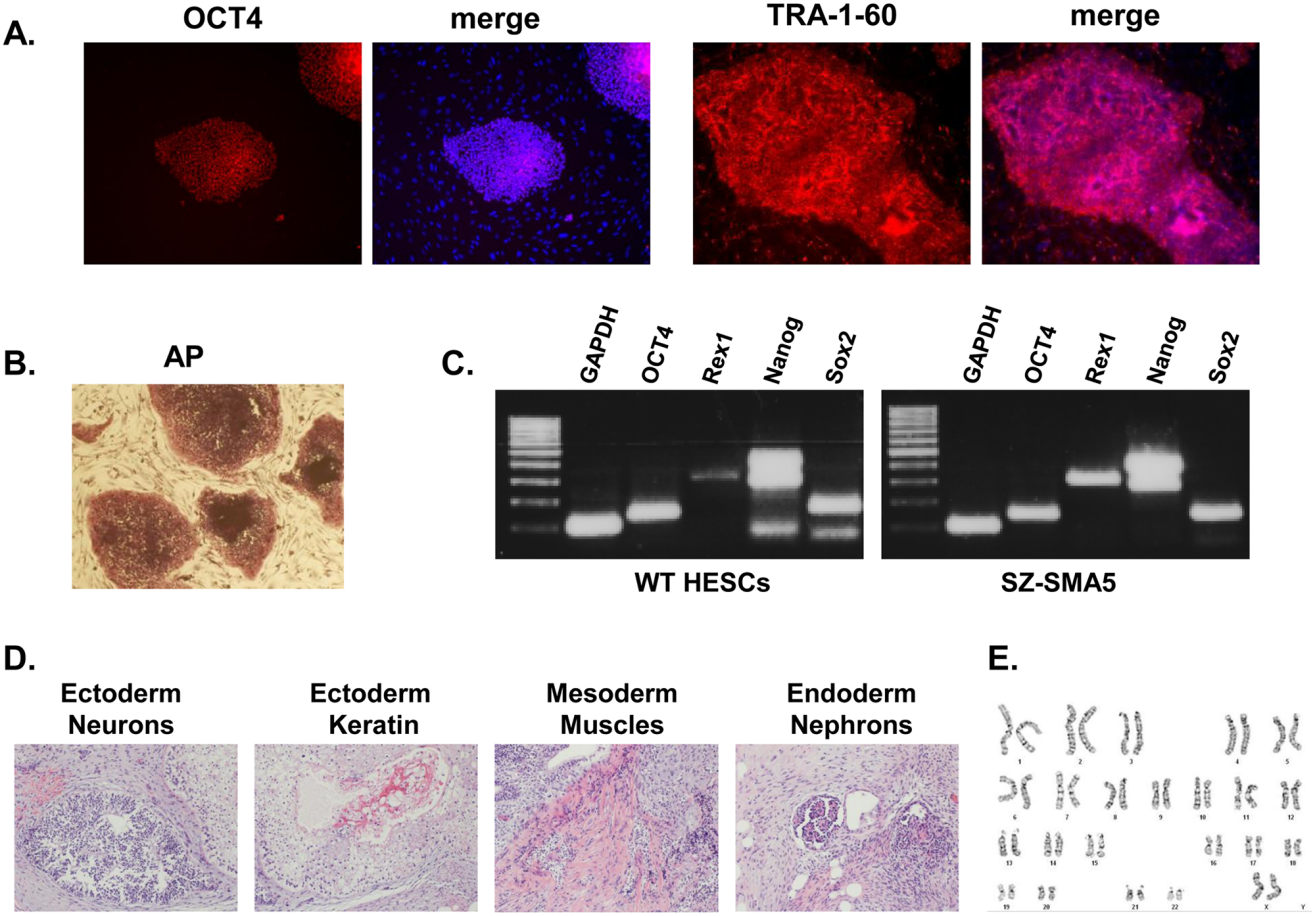
Characterization of SZ-SMA5. (A) Expression of undifferentiated cell specific markers by immunostaining for *OCT4* (red nuclear staining, merged onto Hoechst (blue)), for the cell surface marker *Tra 1–60* (red, merged onto Hoechst (blue)); and (B) for alkaline phosphatase activity (AP). (C) Expression of undifferentiated cell specific markers: *OCT4*, *REX1*, *NANOG* and *SOX2* in SZ-SMA5 and a wild-type (WT) HESC control by RT-PCR. *GAPDH* expression served as a loading control. (D) Teratoma sections stained by H&E from SZ-SMA5 demonstrating multi-cellular structures derived from the three different embryonic germ layers. (E) A representative normal 46(XX) karyotype in SZ-SMA5 as identified by Giemsa staining of 20 different spreads of metaphase chromosomes.

The SMA pHESC line that we established was derived from an embryo, obtained by PGD, to a couple that carries a common deletion of the *SMN1* gene. Following haplotype analysis using 8 tightly linked informative markers, the embryo was typed as potentially affected since it carried the mutant chromosome of the mother, but failed to reveal the alleles of the father. As it had a 50% risk of being affected, it was classified as disqualified for embryo transfer and donated for HESC lines derivation by the parents (IRB 87/07). The newly established cell line (termed SZ-SMA5) displays all key features of pluripotent cells. It has an ES-like cell morphology, is capable of unrestricted growth in culture, and it expresses the typical markers of undifferentiated cells (Fig 1A–1C). In addition, SZ-SMA5 has the potential to differentiate into a wide range of cell types since it can be used to induce teratomas in immune-compromised mice (Fig 1D). In addition, Giemsa staining of 20 different high quality metaphase spreads demonstrated, without exception, a normal diploid 46(XX) karyotype (Fig 1E).

**Fig 2:**
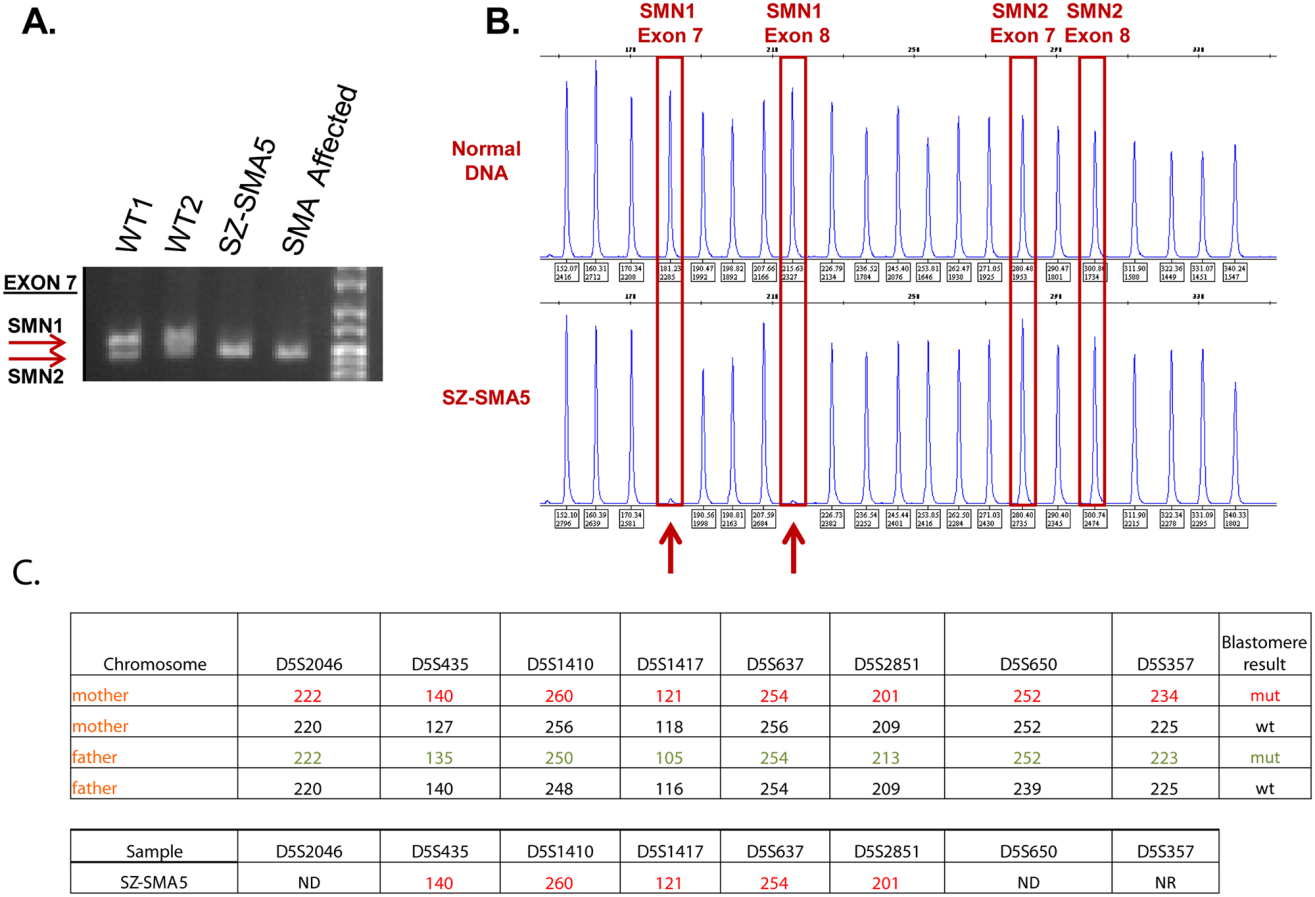
*SMN1* genotyping and haplotype analysis in SZ-SMA5 HESCs. Homozygous loss of *SMN1* was assessed by (A) PCR amplification of exon 7 with a mismatch forward primer that creates a DraI restriction site in *SMN2*, but not in *SMN1*. After DraI restriction digestion, wild-type samples exhibit two bands in this assay while samples completely lacking *SMN1* exhibit only one band. Depicted are two wild-type (WT1 and WT2) HESC lines; SZ-SMA5; and an unrelated SMA-affected HESC line with homozygous deletion of *SMN1* (SZ-SMA6) as a positive control. (B) *SMN1* and *SMN2* copy number was assayed with a commercial MLPA test. Capillary electrophoresis patterns of DNA samples from normal (wild-type) and SZ-SMA5 cells illustrates homozygous deletion of *SMN1* as indicated by the gene-specific absence of PCR products from exons 7 (183 nt) and 8 (218 nt) (marked by red arrows). (C) PCR fragment sizes of *SMN1*-flanking microsatellites in SZ-SMA5 demonstrate that the HESCs were derived from an embryo which inherited a maternal mutation-linked allele, but no paternal allele.

The high similarity between *SMN1* and *SMN2* complicates direct genotyping for SMA diagnosis. To overcome this problem and verify homozygote deletion of *SMN1* in SZ-SMA5, we utilized two established assays. In one assay we PCR amplified exon 7 with a mismatch forward primer that differentially creates a DraI restriction site in *SMN2*, but not *SMN1* [21] such that all exon 7 molecules will be cleaved in the absence of *SMN1* (unlike for homozygote wild type or heterozygote samples in which *SMN1* exon 7 molecules will not be cleaved). In the second assay, we assessed copy number variations in *SMN1* and *SMN2* with a commercial multiplex ligation-dependent probe amplification (MLPA) kit featuring sequence-specific probes that differentiate between copy number changes in *SMN1* and *SMN2*. Using both diagnostic procedures we confirmed the PGD test results for SZ-SMA5 by demonstrating the *SMN1*-specific absence of both exons 7 and 8 (Fig 2A and 2B). In addition, we validated the transmission of parental mutant chromosomes to SZ-SMA5 by carrying out haplotype analysis using 5 gene-flanking informative microsatellite markers for embryo typing. Interestingly, this allele-specific confirmation identified only the mother’s mutant *SMN1* allele. No paternal alleles (wild type or mutant) were identified with the same markers (Fig 2C).

**Fig 3:**
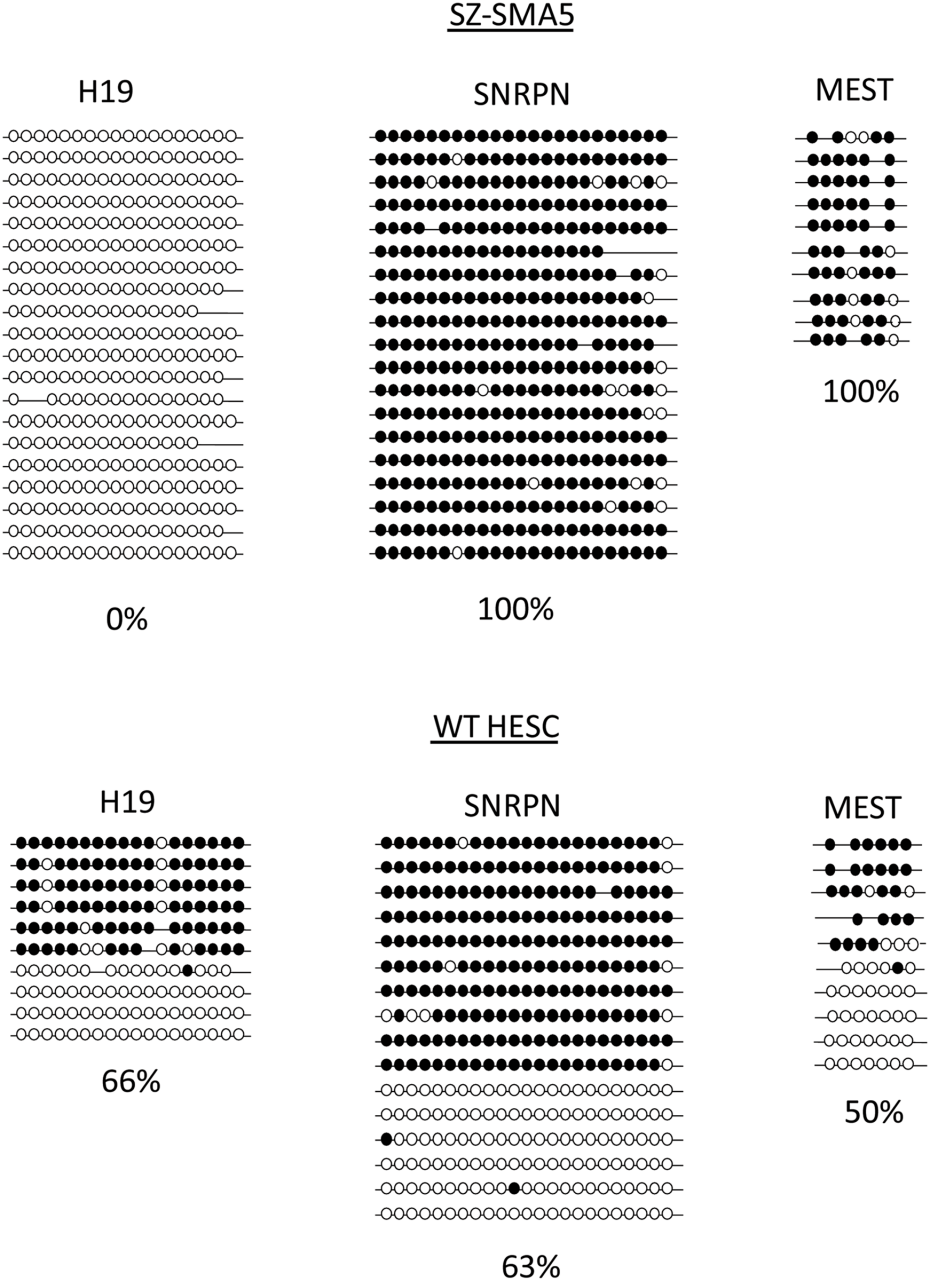
Methylation levels at *H19*, *SNRPN* and *MEST* imprinted loci. Bisulfite single colony sequencing was performed on 3 different imprinted regions (*H19*, SNRPN and *MEST*) to determine methylation levels in normal (WT) and SZ-SMA5 HESC lines. Each line represents a single DNA molecule. Full circles correspond to methylated CpGs while empty circles represent unmethylated CpGs.

To explain the absence of paternal *SMN1*-flanking alleles in an embryo with a 46(XX) karyotype and homozygosity for the *SMN1* deletion, we speculated that either SZ-SMA5 had maternal uniparental isodisomy (UPD) for chromosome 5 (*SMN1* is located in 5q13.2) or alternatively, it may be a product of parthenogenesis. To distinguish between these two genomic states, we determined methylation levels of the cells at three different previously characterized imprinted loci. DNA methylation status was determined for the maternal imprinted loci *SNRPN*, and *MEST* (expected to be differentially methylated when transmitted by the mother) and for the paternal imprinted locus *H19* (expected to be differentially methylated when transmitted by the father). DNA bisulfite colony sequencing showed that, in contrast to normal HESCs (which reveal approximately 50% methylation in all of the aforementioned loci), the *SNRPN* and *MEST* were totally methylated (100%); while the *H19* was completely unmethylated (0%) (Fig 3). These methylation patterns are characteristic to cells that contain maternal but not paternal genomes and are in line with the differential expression of maternal, but not paternal, inherited genes such as *H19* and *SNRPN*, respectively (S1 Fig). Therefore, based on the methylation status of three different imprinted genomic regions and the expression of two oppositely imprinted genes, we hypothesized that SZ-SMA5 was established from a maternal uniparental embryo.

**Fig 4:**
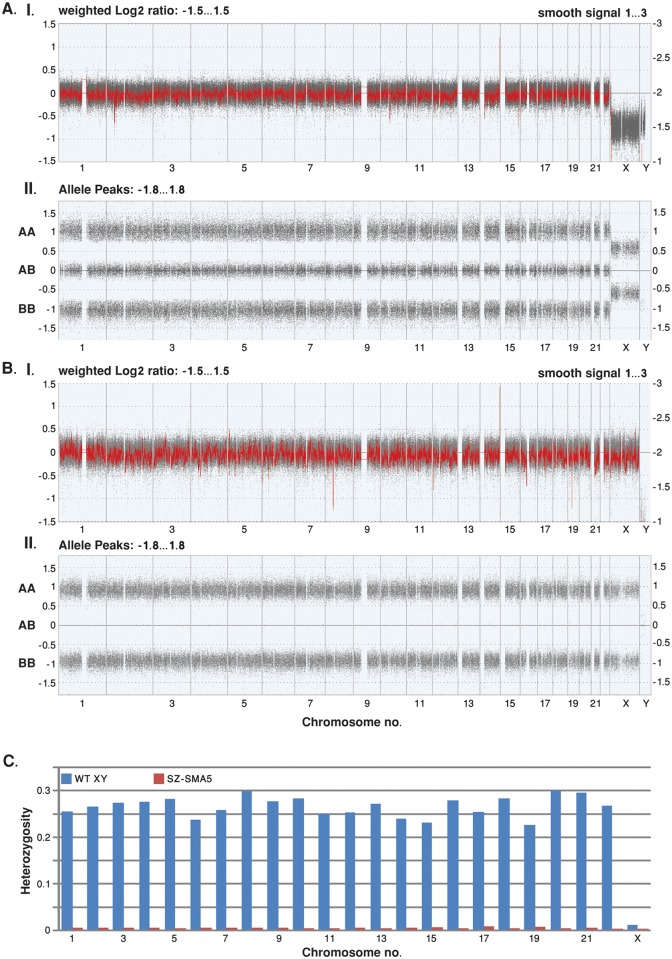
Whole genome view of Cytoscan SNP Array data. Genome wide SNP array results obtained from (A) reference DNA from a male with a normal karyotype (46; XY); and (B) SZ-SMA5 HESC line DNA. The X axis represents chromosomes 1–22, X and Y. (I) The Y axis represents the copy number, determined by the log2 ratio (grey dots) on the left side of the graph, and it's smoothed ratio (red line) on the right. The expected copy number is 2 for autosomal chromosomes (log2 of 0 and smooth signal of 2). The log2 ratio and the smooth signal are determined from both the nonpolymorphic copy number probes and the polymorphic SNP probes. (II) The Y-axis corresponds to homozygote calls (AA or BB) and heterozygote calls (AB). Allele peaks of 1, 0, and -1 indicate a copy number of two, while allele peaks of 0.5 and -0.5 indicate a copy number of one. Allele peaks are calculated from SNP probes. The distinction between XY (reference DNA) and XX (SZ-SMA5) cells is clearly illustrated by the difference in X chromosome copy number. In addition, the overall 0.49% inherent heterozygote call error rate in SZ-SMA5 is below even the expected array genotyping error of ~1% (as determined by dividing the number of heterozygous calls by the total number of SNP probes on the array). Therefore, these data indicate that SZ-SMA5 features a completely homozygous diploid genome. (C) Fraction of SNP heterozygote calls in WT male reference and SZ-SMA5 DNA. Chromosomes are indicated in the X axis and the Y axis indicates the fraction of heterozygous SNP calls per total SNP calls on each chromosome.

To provide an additional assay that distinguishes between maternal UPD and parthenogenesis, we determined the parental identity and degree of heterozygosity of the cell line by genome-wide SNP microarray analysis (Fig 4). Indeed, this comprehensive assessment confirmed homozygosity across the entire genome of SZ-SMA5 (Fig 4A and 4B, S2 Fig and S1 File). Strikingly, this complete absence of heterozygosity (<1%) is evident in each chromosome of SZ-SMA5 as compared with reference DNA (which averages 23%-30% heterozygous SNPs per chromosome; Fig 4C). Therefore, these data unambiguously demonstrate that SZ-SMA5 has originated from a reduplication of a haploid set of chromosomes. This experiment, together with bisulfite sequencing, imprinted gene expression, and haplotype data provides firm evidence that the HESC line in question was in fact derived from a maternal uniparental embryo. Since we could not find any indication for recombination events by SNP array, we conclude that parthenogenesis was achieved through the activation of a mature unfertilized mutant oocyte. Thus, our SMA pHESC line represents a proof-of-principle outcome of mutant oocyte conversion into diploid homozygous mutation-bearing HESCs without need for gene manipulation.

## Discussion

We report on the derivation of a diploid 46(XX) HESC line that is affected with SMA due to homozygosity for a common deletion in the *SMN1* gene. By characterizing the methylation status of three imprinted loci (MEST, PWS- and H19-imprinting centers), and carrying out genome-wide SNP analysis, we show that this cell line was established from a maternal uniparental embryo. Hence, our SMA pHESC line demonstrates how parthenogenesis could be applied as a tool to create HESCs that are homozygote for a disease-causing mutation without intervening with the cell’s genome. The advantage of this would be for disease modelling, being the sole approach for inducing large or complex mutations such as in the *SMN* locus; or other gross DNA alterations such as large unstable repeat expansions or chromosomal rearrangements that otherwise cannot or are difficult to artificially induce (even with the CRISR-Cas9 system). We provide evidence that parthenogenesis was achieved by the activation of a haploid mutant ovum, rather than from Metaphase I (MI) or Metaphase II (MII) stage oocytes, since homozygosity was revealed throughout the entire genome without signs for recombination events. It is difficult to predict when haploid duplication occurred as it may have happened at the 1-cell stage, during preimplantation development or, during the course of cell line derivation/propagation.

Parthenogenesis as a means for homozygotation of naturally inherited defects depends on the accessibility of mutant oocytes. PGD provides an exceptional opportunity to procure and derive such oocytes since it often involves the retrieval of premature oocytes (GV stage) and activated eggs (as in conventional IVF cycles) [23–28]. Clearly, the availability of effective protocols for oocyte maturation and egg activation are anticipated to make this approach widely applicable [29]. One limitation to the proposed system is the uncertainty of the oocyte’s genotype at the time of activation. Hence, it may lead to the unintentional creation of disease-free pHESCs (a 50% chance of being mutant). Although parthenogenesis may result in the creation of mutation-free pHESCs, these cells may provide an unlimited cell source for creating fully-matched cells for disease therapeutics. This latter approach was formerly shown to be feasible in a mouse for correcting beta-thalassemia mutation carriage [30]. In addition, one must consider that if parthenogenesis is achieved by the interruption of meiosis II, rather than from activation of a haploid oocyte, then homozygosity is not guaranteed. This is because the HESCs may present heterozygosity at distal regions that reflect earlier events of recombination [18,19].

There may be several advantages of pHESCs over conventional HESCs. They may be ethically more acceptable since they involve the destruction of embryos that have no real potential to support full term embryonic development. If shown to be safe and effective, they may be favorable over tailor-made patient-derived induced pluripotent stem (iPS) cells or somatic cell nuclear transfer (SCNT)-HESCs for overcoming immunological barriers by allowing the generation of HESC lines with more desirable haplotypes (homozygosity at the HLA and blood group loci) [18,28] [31]. In addition, pHESCs can serve as a useful model system to study the role of genomic imprinting during early embryonic development [32]. Here we provide a proof-of-concept for an additional application; how parthenogenesis can be exploited as a tool for generating homozygous mutant HESCs.

## Supporting Information

S1 Fig Expression pattern of imprinted genes in SZ-SMA5 by RT-PCR. Differential expression of SNRPN (paternally imprinted) but not H19 (maternally imprinted) in SZ-SMA5, is different from wild-type HESC control (WT) and corresponds to the maternal only origin of SZ-SMA5 cell line.
(TIF)

S2 Fig Validation of SNP array results by PCR sequencing. Sequencing results (shaded) of 4 randomly selected probes from the SNP array (rs631376, rs7580488, rs9284754 and rs964944) were used to validate the CytoScan 750K array data obtained for SZ-SMA5. For each probe heterozygosity rate (according to the western european ancestry, CEU), allele variations, and SNP array result are indicated, illustrating high accuracy of the assay for whole genome homozygosity detection.
(TIF)

S1 File Primer lists and PCR conditions. (DOCX)
