# Supplementary figures and images for: Establishment of Homozygote Mutant Human Embryonic Stem Cells by Parthenogenesis
Source: PLoS One. 2015 Oct 16;10(10):e0138893. doi: 10.1371/journal.pone.0138893 (PMC4608834; doi:10.1371/journal.pone.0138893)

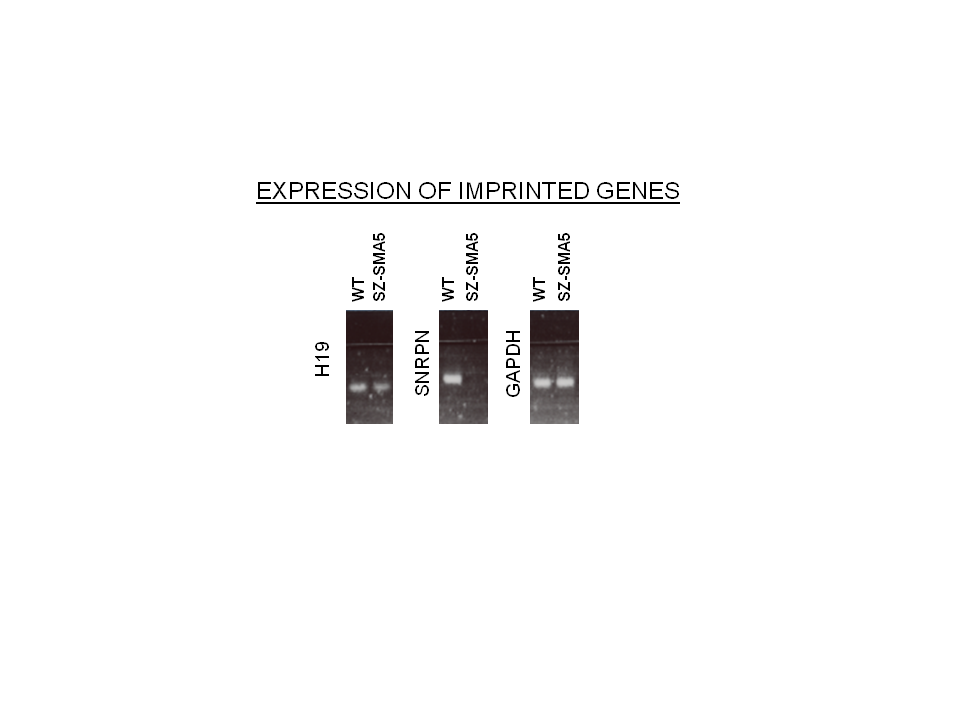

Supplement: S1 Fig — Differential expression of SNRPN (paternally imprinted) but not H19 (maternally imprinted) in SZ-SMA5, is different from wild-type HESC control (WT) and corresponds to the maternal only origin of SZ-SMA5 cell line. (TIF) [file pone.0138893.s001.tif]

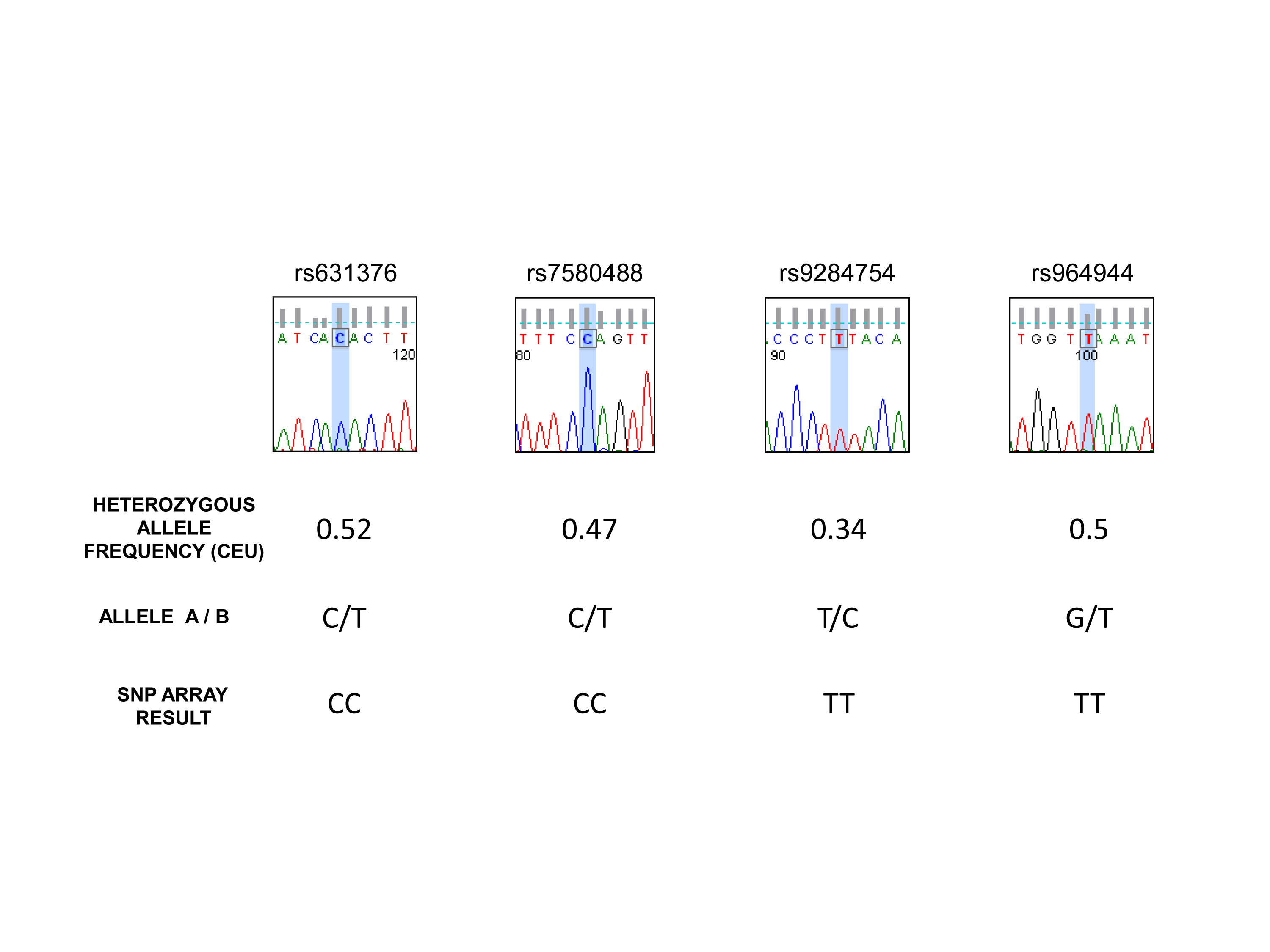

Supplement: S2 Fig — Sequencing results (shaded) of 4 randomly selected probes from the SNP array (rs631376, rs7580488, rs9284754 and rs964944) were used to validate the CytoScan 750K array data obtained for SZ-SMA5. For each probe heterozygosity rate (according to the western european ancestry, CEU), allele variations, and SNP array result are indicated, illustrating high accuracy of the assay for whole genome homozygosity detection. (TIF) [file pone.0138893.s002.tif]
