# Supplementary material for: Establishment of Homozygote Mutant Human Embryonic Stem Cells by Parthenogenesis
Source: PLoS One. 2015 Oct 16;10(10):e0138893. doi: 10.1371/journal.pone.0138893 (PMC4608834; doi:10.1371/journal.pone.0138893)
Supplement: S1 File — (DOCX) [file pone.0138893.s003.docx]

Primer for RT-PCR:

|  | **5' Primer (sequence 5'-3')** | **3' Primer (sequence 5'-3')** | **Annealing Temp ºC** | **Product Size (bp)** |
| --- | --- | --- | --- | --- |
| ***OCT4*** | GACAGGGGGAGGGGAGGAGCTAGG | CTTCCCTCCAACCAGTTGCCCCAAAC | 60 | 144 |
| ***NANOG*** | CAGCCCCGATTCTTCCACCAGTCCC | CGGAGATTCCCAGTCGGGTTCACC | 55 | 342, 390 |
| ***REX1*** | CAGATCCTAAACAGCTCGCAGAAT | GCGTACGCAAATTAAAGTCCAGA | 60 | 306 |
| ***SOX2*** | GGGAAATGGGAGGGGTGCAAAAGAGG | TTGCGTGAGTGTGGATGGGATTGGTG | 55 | 151 |
| ***H19*** | GGAGTTGTGGAGACGGCCTTGAGT | CCAGTCACCCGGCCCAGATGGAG | 62 | 100 |
| ***SNRPN*** | GTCTTCAGAAGCATCAAGTTTTAAC | GCCATCTTGCAGGATACATCTC | 60 | 127 |
| ***GAPDH*** | CCACTCCTCCACCTTTGAC | ACCCTGTTGCTGTAGCCA | 62 | 102 |

Primers for the SMA deletion detection:

|  | **5' Primer (sequence 5'-3')** | **3' Primer (sequence 5'-3')** | **Annealing Temp ºC** | **Product Size (bp)** |
| --- | --- | --- | --- | --- |
| **SMA7F1/R2** | TGCAGCCTAATAATTGTTTTCTTTGGG | GCACCTTCCTTCTTTTTGATTTTGTTT | 60 | 300 |

Primers for haplotype confirmation:

| **Microsatellite** | **5' Primer (sequence 5'-3')** | **3' Primer (sequence 5'-3')** | **Annealing Temp ºC** | **Product Size (bp)** |
| --- | --- | --- | --- | --- |
| **D5S2046** | AAAGCAGAGATTCTGACCCA | GGGAACATCCCATAAAATACTTAGC | 56 | 225 |
| **D5S435** | CAAGAGCACAGTTTGGAGTGAG | ACACACATGCACGCTCTCTC | 60 | 140 |
| **D5S1410** | CTGGTCCACCCCACCTACTA | AGGCTGAGACAGGAGAATCG | 62 | 305 |
| **D5S1417** | TTGCTTCAAAGCAGCTTATATTTTT | CTGGGCGACAGAGTGAGAC | 54 | 270 |
| **D5S637** | TGAATCTCAGGGAGTGTGAA | AAAGGAATCATTACCCCAGA | 56 | 335 |
| **D5S2851** | CCAATAAGTCATGGCAGCTAA | GAGCAAAATGTGCAAACATG | 56 | 240 |
| **D5S650** | ACTGCACACAGCAGCAAGTC | AAAGTCTTTGGGATTTTAATGGAA | 60 | 305 |
| **D5S357** | GTAGAGACAGAGTTTCACCA | GTATCCACTAACCATGAGAA | 56 | 300 |

Primer sets for bisulfite analysis:

|  | 5' primer (sequence 5'-3') | 3' primer (sequence 5'-3') | Annealing Temp ºC | Product size (bp) |
| --- | --- | --- | --- | --- |
| **SNRPN** | TCCAAAACAAAAACTTTAAAACCCAAATTC | AGGTTTTTTTTTATTGTAATAGTGTTGTGGGG | 62 |  |
| **SNRPN**  **nested** | TCAATACTCCAAATCCTAAAAACTTAAAATATC | TGTGGGGTTTTAGGGGTTTAGTAGTTTTTTTTTTTTA | 62 | 341 |
| **H19** | TTTTTGGTAGGTATAGAGTT | AAACCATAACACTAAAACCC | 56 |  |
| **H19 nested** | TGTATAGTATATGGGTATTTTTGGAGGTTT | TCCCATAAATATCCTATTCCCAAATAACC | 62 | 231 |
| **MEST** | GGTGAGATTAGGGTTATTATGGAT | AAAAAAAAATATCACTCCTACCC | 63 | 160 |

Primer sets for homozygosity test:

|  | **5' Primer (sequence 5'-3')** | **3' Primer (sequence 5'-3')** | **Annealing Temp ºC** | **Product Size (bp)** |
| --- | --- | --- | --- | --- |
| **rs631376** | AGGTTTAGGAGTCAGTCGTCAT | TGAGCTGAGATTTGAACCTGG | 60 | 236 |
| **rs7580488** | CCAGCAGGTAGTCAGACAAAG | ACATGTGGCTTGCATTCTATTTC | 60 | 217 |
| **rs9284754** | AGCCATCTGTATCACTGACCA | CTGGGAGAATGGAAGAGGAGA | 60 | 208 |
| **rs964944** | TGGTTCTGGGTCTGTGTTGA | TGGCCTTGGGAATATTGGGA | 60 | 209 |
